# Effectiveness of Outpatient Pulmonary Rehabilitation in Patients with Surgically Resected Lung Cancer: A Retrospective Real-World Analysis

**DOI:** 10.3390/cancers14143479

**Published:** 2022-07-18

**Authors:** Oliver Illini, Arschang Valipour, Dietlinde Gattinger, Milos Petrovic, Hannah Fabikan, Maximilian Johannes Hochmair, Ralf Harun Zwick

**Affiliations:** 1 Department of Respiratory and Critical Care Medicine, Klinik Floridsdorf, Vienna Hospital Association, 1210 Vienna, Austria; arschang.valipour@gesundheitsverbund.at (A.V.); maximilian.hochmair@gesundheitsverbund.at (M.J.H.); 2 Karl Landsteiner Institute for Lung Research and Pulmonary Oncology, Klinik Floridsdorf, 1210 Vienna, Austria; hannah.fabikan@extern.gesundheitsverbund.at; 3 Outpatient Pulmonary Rehabilitation, Therme Wien Med, 1100 Vienna, Austria; dietlinde.gattinger@thermewien.at (D.G.); milos.petrovic@thermewien.at (M.P.); ralf.zwick@thermewienmed.at (R.H.Z.); 4 Ludwig Boltzmann Institute for Rehabilitation Research, 1100 Vienna, Austria

**Keywords:** outpatient pulmonary rehabilitation, lung cancer, real-world data, NSCLC, exercise tolerance, six-minute walk test

## Abstract

**Simple Summary:**

Patients with lung cancer often suffer from low exercise capacity and reduced quality of life. An outpatient pulmonary rehabilitation program may improve exercise performance and reduce symptom load in these patients. We performed an analysis on lung cancer patients after surgery who completed 6 weeks of an outpatient pulmonary rehabilitation. After the rehabilitation program, there was a meaningful improvement in different exercise and strength tests. Patients with surgically resected lung cancer may benefit from an outpatient pulmonary rehabilitation program.

**Abstract:**

Patients with lung cancer frequently suffer from physical deconditioning, low exercise capacity, and reduced quality of life. There is little evidence on the effects of a structured outpatient pulmonary rehabilitation program (OPR) on exercise capacity and symptom load in these patients. We performed a retrospective, single-center analysis of surgically resected lung cancer patients, who underwent a multiprofessional 6-week OPR. The primary endpoint was a change in the six-minute walk test distance (6 MWT). Secondary endpoints included changes in maximal workload and constant work-rate test results during cycle-ergometry, upper and lower extremity strength, and inspiratory muscle strength. The COPD Assessment Test (CAT) was used to assess symptom burden. Fifty-seven patients were included. Of those, fifty-two (91.2%) completed the full 6 weeks of OPR. The mean age was 56.4 (SD 9.2) years, and 58% were female. At completion of OPR, there was a statistically significant mean of a 50 m (95% CI, 29.6–70.7; *p* < 0.001) increase in 6 MWT. Significant improvements were also seen in all other exercise and strength tests (*p* < 0.001), accompanied by a significant reduction in the CAT score (mean difference −3.1, *p* = 0.001). No adverse effects were reported. OPR for surgically resected lung cancer patients was safe and effective and showed high adherence in the current study.

## 1. Introduction

Lung cancer is the most frequently diagnosed cancer and is the leading cause of cancer deaths worldwide [1]. Multimodal therapeutic regimens are used to improve poor prognosis. The consuming character of the disease and debilitating therapeutic procedures such as surgery, chemotherapy, or radiotherapy often cause high symptom burden and loss in functional capacity and quality of life of the patients [2]. In addition, lung cancer patients frequently have cardiac or pulmonary comorbidities that lead to reduced exercise performance and physical activity, muscle weakness, and increased symptoms [3,4,5]. Due to these multifactorial conditions, patients with lung cancer have been shown to have reduced exercise capacity, protein catabolism, and anemia, as well as weight and muscle loss [6,7,8]. Additionally, symptoms such as dyspnea and fatigue can lead to lower physical activity, which results in further muscle loss and further decreases in exercise capacity [2,9].

Pulmonary rehabilitation is a multidisciplinary intervention that aims to reduce functional impairment, symptoms, and disability in people with lung disease. The rehabilitation process consists of physical training, disease education, and nutritional and psychological counseling, along with social and behavioral interventions. For patients with chronic obstructive pulmonary disease (COPD), pulmonary rehabilitation is widely known to be an effective intervention to improve exercise performance, dyspnea and quality of life [4]. It is recommended by national and international guidelines as an important component in the management of COPD [10,11]. For those chronic lung diseases, it is also proven that outpatient pulmonary rehabilitation programs (OPR) can be an effective and useful way to combine advantages of intense rehabilitation procedures while enabling patients to spend minimal time at healthcare institutions [4].

There is increasing evidence that pulmonary rehabilitation can also improve a variety of meaningful outcomes in patients with lung cancer (especially following lung resection), such as exercise performance, quality of life, fatigue, and dyspnea [12,13,14,15,16,17], and it is widely agreed that rehabilitation is needed across all stages of the disease [18]. However, the specific role of pulmonary rehabilitation in the clinical management of lung cancer patients is still unclear. It is still under debate if, when, and in which form rehabilitation procedures can be implemented in the oncologic treatment course and which patients are likely to benefit. Additionally, there is no standardization of the program components. Furthermore, data on OPR for lung cancer patients are lacking.

The main objective of this retrospective analysis was to evaluate efficacy, safety, and adherence of OPR in lung cancer patients under real-world conditions.

## 2. Methods

### 2.1. Study Design

This was a retrospective, single-center, real-world analysis of all consecutive patients (age ≥18 years) with histologically confirmed surgically resected lung cancer who were referred to an OPR referral institution in Vienna (Therme Wien Med, Vienna, Austria) between July 2012 and August 2019. For the comparison of the primary outcome, all consecutive patients with COPD who underwent the same OPR between April 2013 and October 2019 were analyzed.

### 2.2. Ethics Approval and Informed Consent

The study protocol was approved by the ethics committee of the city of Vienna, Austria (EK 21-258-VK, 22.11.2021). All patients provided written informed consent for analysis of their rehabilitation data. The study was conducted according to the principles of the Declaration of Helsinki.

### 2.3. Rehabilitation Procedures

At the beginning of OPR, detailed information about the medical history and demographics have been evaluated, including tumor stage, histology, comorbidities, and medication. Inclusion of patients to OPR and individualized rehabilitation procedures were conducted according to the Austrian guidelines for OPR [19] and were executed by a multiprofessional team. Participants practiced endurance, strength, and inspiratory muscle training for 3–4 h, three days a week (see Supplementary S1 for detailed description). Rehabilitation was considered successfully completed after completion of at least six consecutive weeks of OPR.

### 2.4. Outcome Variables

The primary outcome was defined as the change in six-minute walk test in meters (6 MWT) after the completion of OPR. It was assessed according to the standardized protocol of the American Thoracic Society Guidelines for the six-minute walk test [20]. A clinically relevant change (minimal clinical important difference, MCID) in 6 MWT was defined as a minimum of 30.5 m according to a systematic review [21].

Secondary outcome variables concerning physical exercise were changes in maximal workload in watts that patients achieved on cycle-ergometer (Wmax), constant work-rate test at 70% of Wmax in minutes (CWR70%), upper-extremity strength in kilograms (UE), lower-extremity strength in kilograms (LE), and maximal inspiratory-muscle strength in millibars (PiMax). Additionally, lung-function measurements of forced expiratory volume in one second (FEV1) and residual lung volume in liters (RV) were performed. The “COPD Assessment Test” (CAT) was used to assess symptom burden. The MCID for CAT was defined as a change of 2 points [22]. The MCID for Wmax was defined as +6.8 W, and for CWR70% as +1.75 min [23]. All parameters were assessed at the beginning and termination of OPR.

### 2.5. Statistical Analysis

Descriptive categorical data are expressed as frequencies and proportions, and continuous data, as mean ± standard deviation (SD). For changes in 6 MWT and in secondary outcome parameters, the differences between pre- and postrehabilitation values were assessed using one-sample Student’s t-tests for paired data in case of normality of the distribution of mean differences. In case of abnormal distribution of mean differences, Wilcoxon’s paired rank sum test was used. Mann-Whitney U Test was used for comparison of the study population and the COPD control group. Two-tailed tests were performed in all samples with a level of significance of 5%. Normality of distribution was assessed by Shapiro-Wilk test and graphically by histograms and Q-Q-plots. All statistical analyses were conducted using SPSS version 26 (IBM, Armonk, NY, USA).

## 3. Results

### 3.1. Study Population and Adherence

Fifty-seven patients with histologically confirmed and surgically resected lung cancer were referred to OPR between July 2012 and August 2019. In five cases (8.8%), rehabilitation procedures were stopped before completing the required 6 weeks (four patients because of medical reasons related to oncologic disease and one patient decided to stop prematurely due to personal reasons). Rehabilitation was successfully completed by 52 patients (91.2%). Of those, 27 patients completed 100% of their scheduled rehabilitation sessions, 17 completed at least 90%, 5 at least 80%, and 5 at least 50%. No adverse events were reported. The clinical characteristics of patients are summarized in Table 1.

Of the patients who completed OPR, 30 were female (58%). The mean age was 56.4 (SD 9.2) years. Most patients (*n* = 44, 84.6%) had non-small-cell lung cancer (see Table 1 for the histologic subtypes). Neuro-endocrine carcinoma (NEC) was found in eight patients (15.4%), of which five had a carcinoid and three, a NEC grade III tumor. Tumor stages ranged from IA to IIIA (Staging per UICC Version 8). Most patients were referred in earlier tumor stages, with 30 (61.2%) in stage I, 5 (10.2%) in stage II, and 14 (28.6%) in stage III, respectively. The tumor stage was unknown for three patients (5.8%). All patients underwent surgery for lung cancer with curative intention before being referred to OPR. Of those, additional information about the procedure of surgery could be collected in 40 patients. Lobectomy was the most common procedure (*n* = 34, 85%) followed by pneumectomy (*n* = 4, 10%) and segmental resection (*n* = 2, 5%). Twenty-four patients (54.5%) received chemotherapy before being referred to OPR, either in a neoadjuvant or adjuvant setting. In eight cases (15.4%), information on systemic therapy could not be obtained.

### 3.2. Changes in Outcome Variables

At baseline, the walking distance ranged from 150 to 630 m, with a mean of 472.8 (SD 94.3) meters (Table 2). Assessment of the change in 6 MWT was not possible in eight patients because of a lack of data. After OPR, 36 patients (82%) were able to improve their maximum walk distance. The mean distance at discharge increased to 522.9 (SD 91.5) meters, resulting in a statistically significant (*p* < 0.001) mean improvement of 50.2 m (+10.6%). Twenty-seven patients (61%) showed an improvement in 6 MWT ≥ 30.5 m, thus being considered responders with respect to the threshold of a clinically relevant change. To compare efficacy of OPR in lung cancer patients with the efficacy in COPD, 569 consecutive COPD patients who underwent the same OPR were analyzed for change in distance walked in 6 MWT. The mean age of COPD patients was 51.3 (SD 8.9) years, 59% were male, mean BMI was 26.7 kg/m^2^, mean FEV1 was 1.7 L, and mean FEV1/FVC (ratio of FEV1 to the forced vital capacity) was 54%. Patients with COPD achieved a mean improvement of 34.0 (+7.5%) meters from a mean walking distance of 451.9 (SD 117.3) meters at admission to 585.9 (SD 117.2) meters at discharge. This difference was statistically significant (*p* < 0.001). A Mann–Whitney U Test was calculated to determine if there were differences in changes in 6 MWT pre- to post-OPR between COPD and lung cancer patients. There was no statistically significant difference between those groups regarding change in 6 MWT after OPR (*p* = 0.352).

The values and changes for secondary assessed physical-exercise and lung-function tests are presented in Table 2 and Table 3. All five physical-exercise parameters showed significant improvements at the completion of OPR. The peak performance of Wmax during cycle ergometry increased from 100.9 (SD 31.6) to 115.9 (32.5) W (mean difference 14.6 W; *p* < 0.001). Endurance assessment in terms of CWR70% increased from 9.6 (SD 7.4) to 14.0 (SD 8.6) minutes, which resembles an improvement of 45.8% (*p* < 0.001). The inspiratory muscle strength determined by PiMax was improved by 38 patients (88.4%), with a mean improvement of 17.5 mbar (+21.2%; from 82.7 (SD 34.9) to 100.2 (SD 34.0) mbar; *p* < 0.001).

The strength of the upper and lower extremities was significantly (*p* < 0.001) improved at discharge from 22.0 (SD 8.8) to 28.6 (SD 9.2) kg (mean difference +6.7 kg), and from 107.7 (SD 32.2) to 131.3 (SD 33.1) kg (mean difference +23.6 kg), respectively. Physiological improvements of endurance and strength were paralleled by a reduction in symptom load with a significant (*p* = 0.001) reduction in the CAT score results (Table 2, mean score 14.6 (SD 7.7) lowered to 11.5 (SD 7.5) post OPR; mean difference −3.1 points). A clinically important improvement of at least two points was achieved by 71% of patients.

Lung-function parameters and body mass index at admission and discharge are shown in Table 3. No significant changes could be observed.

## 4. Discussion

We report on 57 patients with surgically resected lung cancer who underwent OPR in a real-world setting with overall high adherence and beneficial outcomes in most patients included in this analysis.

There is growing evidence that pulmonary rehabilitation can be an effective therapeutic intervention in the management of lung cancer patients [12]. Most guidelines, however, do not specify the rehabilitation process and lack guidance with respect to the actual implementation into overall oncologic care [18]. To the best of our knowledge, only few studies address safety, adherence, and efficacy of outpatient rehabilitation in this specific patient population [24,25,26].

In our analysis, OPR was able to increase the distance walked in six minutes in the vast majority of patients. While there are several modalities for the objective evaluation of functional exercise, the 6 MWT is one of the most well-established instruments to measure exercise capacity in patients with pulmonary disease. The distance a patient covers in the predefined time is used as the outcome by which changes in performance capacity can be assessed. As a submaximal self-paced exercise test, the 6 MWT is suitable to assess aerobic capacity and endurance and may reflect the functional exercise level of patients in everyday life [20]. In patients with COPD, it can be a predictive marker for mortality [27] and there is some evidence that results of 6 MWT are an independent prognostic factor for NSCLC patients treated with surgery [28]. Our findings are in line with most of the previously published studies on rehabilitation in lung cancer patients pre- and postsurgery, as well as in inoperable lung cancer patients [13,17,29,30,31,32,33], but data on efficacy in an outpatient setting are rare.

A pilot study with ten lung cancer patients after surgery showed that peak exercise capacity and 6 min walking distance could be significantly improved by an 8-week inpatient rehabilitation program [32]. Between 2001 and 2005, Cesario et al. investigated patients after lung resection for NSCLC completing a 4-week inpatient pulmonary rehabilitation [17]. The 25 patients in the intervention group had a statistically significant improvement in the median distance of 6 MWT from 298 to 393 m. In another randomized controlled trial in patients with resectable lung cancer after thoracotomy, patients were randomized to a 12-week hospital-based rehabilitation program (starting 4 weeks after discharge from hospital) or usual care [29]. Despite no difference found in quality of life—the primary outcome of their study—the 27 patients in the intervention group significantly improved their mean walking distance in 6 MWT from 524 at baseline to 567 m. Compared with the control group, there was a significant improvement in the 6 MWT after 3 months in the intervention group (mean difference between groups 94 (SD 38) meters; *p* = 0.024). In that study, however, high dropout rates in the intervention group observed, mostly explained by pain and due to systemic side effects from adjuvant chemotherapy. The feasibility and adherence of a rehabilitation program in patients with newly diagnosed incurable lung cancer was examined by Temel and colleagues [33]. Twenty-five patients were enrolled, of which only 4% completed 16 supervised interventions during a 12-week hospital-based outpatient exercise program. In this population, a non-statistically significant increase in the 6 MWT from mean 411 to 436 m was observed. The authors acknowledged difficulties with recruitment in their report and suggested that community-based outpatient exercise interventions may potentially have achieved a higher enrollment, as some patients may have concerns with commuting to the hospital. Olivier et al. conducted a prospective observational study of home-based pulmonary rehabilitation in patients with advanced-stage thoracic malignancy, receiving chemotherapy at the time of inclusion: Of 243 eligible patients that were screened, only 71 started the program, with a completion rate of 66% [24]. An improvement in physical activity and anxiety scores were observed following rehabilitation, but there were no improvements in the 6 MWT.

We showed that OPR was able to improve the results in 6MWT in 82% of our patients, with a statistically significant median improvement of 50 m. A minimal clinically significant change was achieved by 61% of patients. Due to its retrospective nature, there was no control group of lung cancer patients without rehabilitation to directly compare the results. However, pulmonary rehabilitation is known and proven to be an effective intervention for patients with COPD. In the Cochrane review (participants = 1879; studies = 38) the median improvement after rehabilitation in patients with COPD in the six-minute walk distance was 43.9 m (95% CI, 32.64–55.21), which is in line with the improvements in our group of lung cancer patients [4]. Furthermore, we compared the improvement in 6 MWT of our lung cancer patients with 569 COPD patients who underwent the same OPR. Lung cancer patients achieved a numerically even higher mean improvement (50 versus 34 m, not statistically significant) than COPD patients, indicating a similar effectivity in these populations. Furthermore, both bicycle-exercise parameters in our collective were significantly improved and clinically meaningful. In addition, we observed a significant improvement in the strength of the upper and lower extremities following OPR, an observation that is in line with previously published reports [30].

OPR was further able to reduce symptom burden in our patient population (Table 2). Symptoms such as cough, dyspnea, and restrictions in daily life improved in most lung cancer patients following OPR. Our findings are in line with previous published study results describing a significant change in Borg scale for dyspnea compared to a control group following an inpatient pulmonary rehabilitation trial, including patients after lung resection for NSCLC [17]. Glattki et al. similarly demonstrated improvements in dyspnea (using the modified Medical Research Council Dyspnea Scale) in 47 NSCLC patients following an inpatient multidisciplinary pulmonary rehabilitation program [31]. The significant influence of OPR in lung cancer patients on other symptoms such as fatigue, anxiety, and overall quality of life has also been described before [13,24,26].

In our analysis, OPR was able to achieve high rates of adherence and patient compliance. A total of 91% of our patients completed the six consecutive weeks of OPR. Of these, 49 (94%) attended at more than 80% of their scheduled rehabilitation sessions. This may be explained by the comprehensive and multiprofessional outpatient rehabilitation program, high motivation of patients after receiving curative surgery, and an adequate selection of patients who were referred to OPR by the treating physicians. The content, setup, and lengths of the rehabilitation procedure might be crucial for patients’ adherence. In the presented study, OPR was conducted over 6 weeks and according to the national guidelines [19], including endurance, strength, and inspiratory muscle training, as well as medical treatment, educational seminars, and nutritional and psychological counseling (see Supplementary S1). A multidisciplinary pulmonary rehabilitation (usually a 3-week inpatient setting) is an obligatory procedure in Austria as well as in some other European countries, e.g., Germany or Switzerland. Several studies have shown that such a comprehensive program can improve quality of life and physical condition in patients with chronic respiratory diseases [34,35,36]. In Austria, reimbursement of pulmonary rehabilitation costs is assured for patients with chronic respiratory diseases or lung cancer. In addition, the fact that our patients did not receive chemotherapy during the OPR period might have contributed to the high adherence. Cavalheri and colleagues conducted a randomized controlled pilot trial concerning supervised exercise training in NSCLC patients after curative treatment [37]. They defined adherence to exercise training as a completion rate ≥60% of training sessions and achieved only 44% adherence in the intervention group. Adherence in other studies is greatly varying, with a trend of higher adherence in an outpatient setting and when patients did not receive chemotherapy during the rehab intervention period [24,26,29,30,33,37,38].

This retrospective analysis has several limitations, including reporting bias, selection bias, and information bias. Furthermore, the outcomes were described within the limitation of a small sample size and there were no patients included with metastatic disease or undergoing chemotherapy during the OPR, so it remains unsure if those patients would have the same potential for OPR. Effects of smoking history and chronic comorbidities (e.g., COPD) that might have influenced the outcome were not taken into account. In addition, the performance status of patients was not systematically evaluated, so it remains uncertain if patients with poor general condition would benefit from OPR. Additionally, we did not observe any improvements in lung-function test results after rehabilitation. These findings are, however, consistent with the large majority of previously published studies and are considered of minor importance, given the lack of a relevant relationship between quality of life and objective lung-function test results in lung cancer patients [39].

## 5. Conclusions

In the current study, OPR seems to be an effective and safe intervention with high adherence for surgically treated lung cancer patients that can be implemented in the therapeutic course. Improvements in functional exercise capacities as well as symptom burden may help in improving patient’s overall quality of life and outcomes. Large controlled trials are urgently needed to determine the exact role of rehabilitation in lung cancer management and answering questions about the right timing, duration, and character of the rehabilitation process. Furthermore, it would be of interest to analyze subgroups of lung cancer patients (e.g., advanced stages vs. early stages or following chemo- or immunotherapy) to determine which training or educational interventions achieve the most beneficial results in a specific subset of patients.

## Figures and Tables

**Table 1:** Patient characteristics.

Patient Characteristic †	Mean (SD), Number (%)	Available Data/Total Number
**Age**		52/52
Age (years)	56.4 (9.2)	
Range (years)	37–76	
**Sex**		52/52
female	30 (58%)	
male	22 (42%)	
**Body weight**		51/52
BMI (kg/m^2^)	27.3 (5.3)	
Current smoker	11 (21%)	52/52
**Tumor Stage** **^‡^**		49/52
Stage I	30 (61.2%)	
Stage II	5 (10.2%)	
Stage III	14 (28.6%)	
Stage IV	0 (0%)	
Range	IA—IIIA	
**Histology**		52/52
**NSCLC**	44 (84.6%)	
Adenocarcinoma	33 (63.5%)	
Squamous-cell carcinoma	5 (9.6%)	
Adeno-squamous carcinoma	1 (1.9%)	
NSCLC-NOS	5 (9.6%)	
**Neuro-endocrine tumor**	8 (15.4%)	
SCLC	1 (1.9%)	
LC-NEC III	2 (3.8%)	
Carcinoid tumor	5 (9.6%)	
**Prior oncologic therapy**		44/52 52/52
Chemotherapy	24 (54.5%)	
Surgery	52 (100%)	

BMI, body mass index; NSCLC, non-small-cell lung cancer; NSCLC-NOS, non-small-cell lung cancer not otherwise specified; SCLC, small-cell lung cancer; LC-NEC III, large-cell neuroendocrine carcinoma grade III; ^†^ percentage may not be 100 because of rounding; ^‡^ staging per UICC Version 8.

**Table 2:** Exercise assessment and CAT at admission and discharge of outpatient pulmonary rehabilitation.

Assessment	Number of Patients	Admission Mean (SD)	Discharge Mean (SD)	Difference	MCID	*p*-Value
6-MWT (m)	44	472.8 (94.3)	522.9 (91.5)	+50.2 (10.6%)	61%	<0.001
Wmax (W)	43	100.9 (31.6)	115.9 (32.5)	+14.6 (14.0%)	66%	<0.001
CWR70% (min)	40	9.6 (7.4)	14.0 (8.6)	+4.4 (45.8%)	65%	<0.001
CAT (score)	24	14.6 (7.7)	11.5 (7.5)	−3.1 (21.2%)	71%	0.001

MCID, ratio of patients who achieved the minimal clinical important difference; 6-MWT—6 min walking test, MCID = +30.5 m; Wmax—maximal workload on cycle ergometer, MCID = +6.8 W; CWR70%—constant work rate test at 70% of Wmax, MCID = +1.75 min; CAT—COPD Assessment Test, MCID = −2.

**Table 3:** Lung function, strength, and BMI assessment at admission and discharge of outpatient pulmonary rehabilitation.

Assessment	Number of Patients	Admission Mean (SD)	Discharge Mean (SD)	Difference (%)	*p*-Value
PiMax (mbar)	43	82.7 (34.9)	100.2 (34.0)	+17.5 (21.2%)	<0.001
UE (kg)	33	22.0 (8.5)	28.6 (9.2)	+6.7 (30.5%)	<0.001
LE (kg)	32	107.7 (32.2)	131.3 (33.1)	+23.6 (22.3%)	<0.001
FEV1 (L)	46	2.1 (0.6)	2.0 (0.5)	−0.1 (4.8%)	0.090
FEV1%FVC	46	71.6 (9.0)	70.8 (8.8)	−0.8 (1.1%)	0.394
RV (L)	44	2.6 (0.8)	2.5 (0.7)	−0.1 (3.8%)	0.471
BMI (kg/m^2^)	51	27.3 (5.3)	27.4 (5.3)	+0.1 (0.3%)	0.631

PiMax, maximal inspiratory-muscle strength; UE, upper-extremity strength; LE, lower-extremity strength. FEV1, forced expiratory volume in one second; FEV1%FVC, ratio of the forced expiratory volume in one second to the forced vital capacity; RV, residual lung volume; BMI, body mass index.

## Data Availability

The data that support the findings of this study are available from the corresponding author upon reasonable request.

## Supplementary Materials

The following supporting information can be downloaded at: https://www.mdpi.com/article/10.3390/cancers14143479/s1, S1: Outpatient pulmonary rehabilitation program.
