# Supplementary material for: Effectiveness of Outpatient Pulmonary Rehabilitation in Patients with Surgically Resected Lung Cancer: A Retrospective Real-World Analysis
Source: Cancers (Basel). 2022 Jul 18;14(14):3479. doi: 10.3390/cancers14143479 (PMC9322504; doi:10.3390/cancers14143479)
Supplement: Supplementary file 1 [file cancers-14-03479-s001.zip › cancers-1761809-supplementary.pdf]

Supplementary Materials

Effectiveness of Outpatient Pulmonary Rehabilitation in Patients with Surgically Resected Lung Cancer: A Retrospective Real-World Analysis

This Supplementary Materials section has been provided by the authors to give readers additional information about their work.

S1. Outpatient pulmonary rehabilitation program

Patients underwent a comprehensive interdisciplinary and individualized rehabilitation program according to the Austrian guidelines for outpatient pulmonary rehabilitation (OPR) that has been described before in detail (Vonbank et al, 2015 [19]). Over a period of 6 weeks patients completed a maximum of 60 rehabilitation sessions of 50 min each. Forty-six of the 60 sessions were training sessions only, which accounts for a net workout time of approximately 38 h. The exercise sessions consisting of endurance, strength and inspiratory muscle training were performed under supervision of medical staff, physiotherapists and sport scientists. Endurance training implemented cycle ergometer exercises targeting a heart rate calculated by the Karvonen formula, strength training included 4-6 muscle groups including upper and lower extremity. All patients received inspiratory respiratory muscle training using a special training device (Eumedics GmbH, Vienna, Austria -RESPIFIT S). Physiotherapy sessions focused on improvement of pulmonary problems, mobility and coordination. Additionally, the program consisted of individualized patient education, which included 4 disease- and medical treatment-specific sessions, 2 sessions focusing on nutritional counseling, and 4 psychologic sessions. If indicated further psycho-social counseling and smoking cessation sessions were offered to all participants. Medical evaluation and assessments at admission and discharge of OPR accounted for another 4 sessions. Those assessments included echocardiography, lung function (spirometry, lung volumes), maximal inspiratory pressure (PiMax), cycle ergometer testing including minimal and maximal heart rate, blood pressure and maximal workload (Wmax), constant work rate test at 70% of Wmax (CW70%), 6-min walk test (6MWT), COPD Assessment Test (CAT) scale, 1-minute sit-to-stand test (1-MSTST), upper and lower extremity strength in kilogram (kg).
